# Obliquely Deposited Gold Nanohelices on Lithography-Free Prepared Nanoseeded Surfaces

**DOI:** 10.1186/s11671-017-2261-z

**Published:** 2017-08-10

**Authors:** Yi-Jun Jen, Wei-Chih Liu, Chih-Yung Hsiao, Po-Cheng Lin, Chia-Liang Yu, Teh-Li Chan

**Affiliations:** Department of Electro-Optical Engineering, National Taipei University of Technology, 10608 No. 1, Sec. 3, Chung-Hsiao E. Rd., Taipei, Taiwan

**Keywords:** Glancing angle deposition, Metal nanohelix, Circular dichroism

## Abstract

A substrate surface on which gold particles are distributed is prepared by annealing an ultra-thin gold film to enable glancing angle deposition. By cooling the substrate and controlling its spin rate, two spiral-like and one screw-like gold nanohelix arrays are grown upon the seeded surfaces. The mean helix radius and pitch length are reduced to 17 and 55 nm, respectively. The g-factor of the three nanohelix arrays is measured here and associated circular dichroism peak blue shifts occur as the gold helices shrink.

## Background

Subwavelength plasmonic helical arrays have been intensively studied over the last 10 years [1]. The circular polarization-dependent absorption and radiation cause the arrays to exhibit extraordinary optical properties, including broadband circular polarization [2] and light absorption [3]. The circular dichroism of plasmonic nanohelices is an important characteristic in bio-sensing [4]. In 2005, three-dimensional gold helices with pitch length of approximately 0.75 μm were fabricated and regularly distributed on a surface by laser writing [2]. Such a regular helix array acts as a circular polarizer that passes right-handed circular waves and blocks left-handed circular waves with wavelengths in the range from 3 to 6.5 μm [2, 5].

Owing to the development of nanotechnology, metal nanohelices with mean pitch length of less than 200 nm have recently been developed by glancing angle deposition [6]. Nanostructured metal films have been sculptured by tilting the substrate during deposition to yield the shadowing effect [7]. Peer Fischer et al. adopted two strategies to realize sub-wavelength three-dimensional structures [8]. The first involves arranging a seeded surface to offer the shadowing effect [9]. The other involves using liquid nitrogen to cool the substrate to around 140 °C to reduce the diffusion energy of adatoms [10]. Two-turn gold nanohelices with mean pitch length of 34 nm and helix radius of 30 nm have been sculptured on a regular seeded surface that had been patterned by lithography. Recently, nanohelices were successfully grown on a smooth surface by self-shadowing effect [11]. Spiral-like or screw-like metal helices were grown by tuning the spin rate of substrate relative to the deposition rate [12, 13]. However, the self-shadowing effect limited the mean size of the nanohelices. At a deposition angle of 89° between the direction of the deposition flux and the surface normal, a silver spiral nanohelix array with mean pitch length (*p*) of 153 nm and helix radius (*R*) of 88 nm and a gold nanohelix array with *p* = 162 nm and *R* = 78 nm were grown on a smooth BK7 substrate.

To form smaller nanohelices than those grown by self-shadowing, a seeded surface is required to tune their morphology [14]. However, the use of expensive lithography to pattern the substrate surface does not provide the advantage of glancing angle deposition [15], which is a cheap method for the mass production of nanohelices. In this work, gold particles were distributed on a substrate surface by annealing an ultra-thin metal film. These particles offer shadowing effect and reduce the size of the gold helices that grow on them [16, 17].

## Methods

**Fig. 1:**
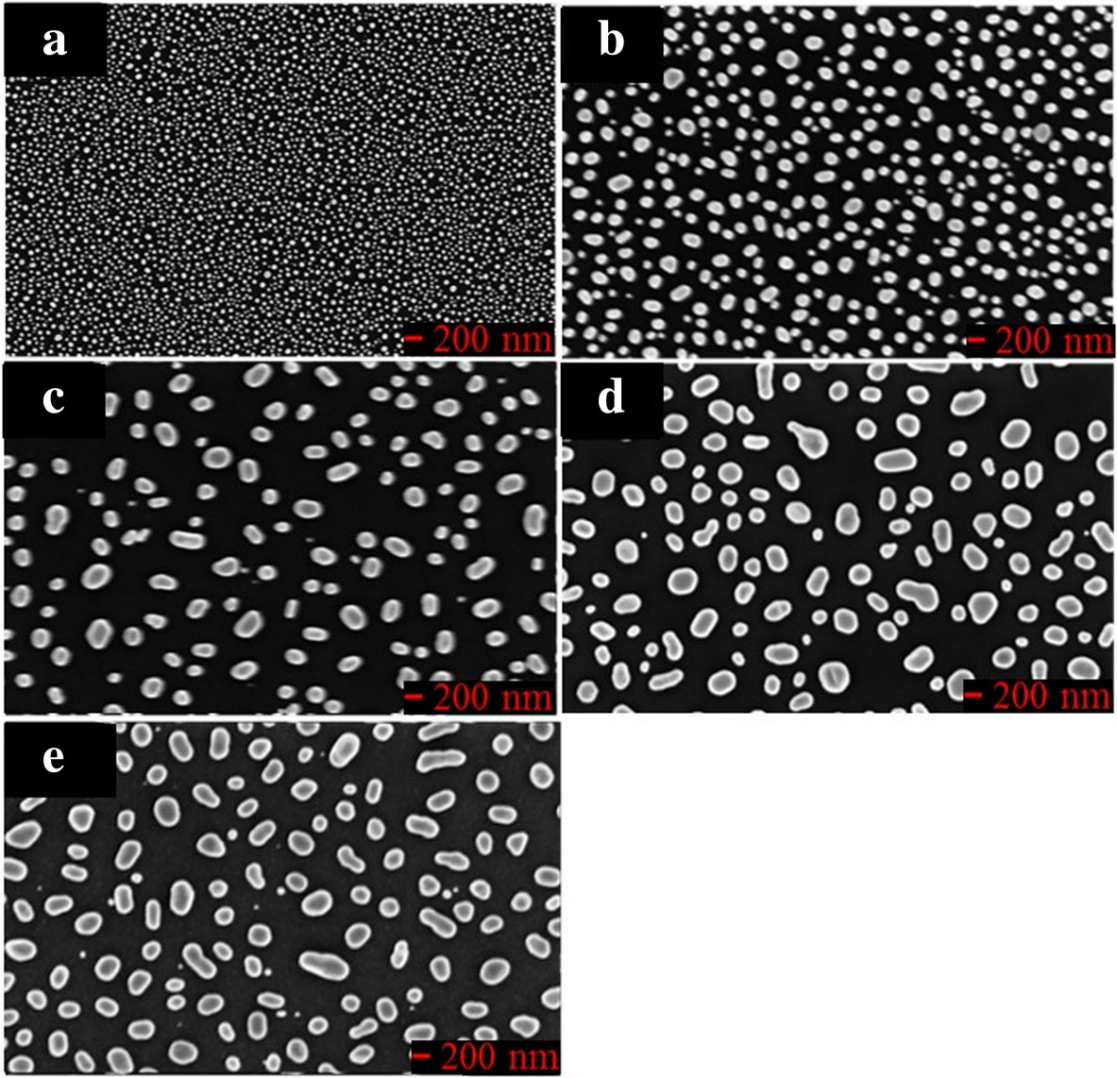
Top-view SEM images of Au particles on surfaces with different mean particle diameters and spacing: **a** (d, s) = (45 nm, 40 nm); **b** (d, s) = (105 nm, 85 nm); **c** (d, s) = (150 nm, 125 nm); **d** (d, s) = (180 nm, 150 nm); **e** (d, s) = (200 nm, 170 nm)

**Fig. 2:**
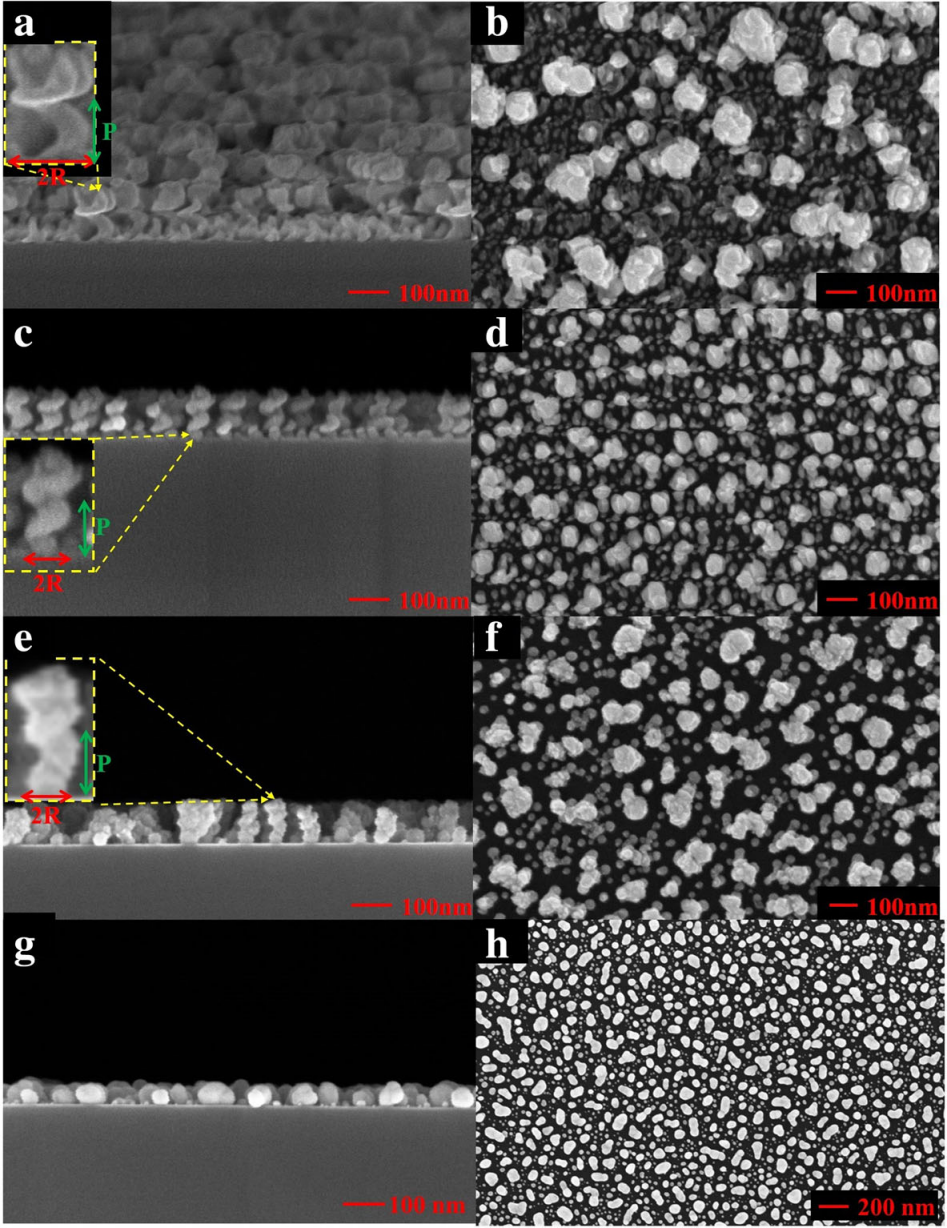
Top-view and cross-section SEM images of 2-turn Au nanohelices deposited at 0.088 rpm (**a**, **b**), 0.117 rpm (**c**, **d**) and 0.160 rpm (**e**, **f**). Nanohelices grown on a smooth surface are shown in **g** and **h**

The substrate was coated with a thin gold film to yield gold nanoparticles on its surface after annealing. Gold films with thicknesses of 5, 10, 15, 20, and 25 nm were prepared by electron beam evaporation. The thickness of each film was controlled by varying the time of deposition and the deposition rate and was measured using a quartz crystal thickness monitor. The mean particle size was controlled by varying the thickness of the initial deposited gold film. Nanoparticles were obtained by annealing the deposited films at 500 °C for 30 min. The mean particle size (*d*) increased from 45 to 200 nm, and the mean spacing(s) between adjacent particles increased from 40 to 170 nm as the initial film thickness increased from 5 to 25 nm, as shown in Fig. 1. In this work, a sample with mean diameter of 45 nm and spacing of 40 nm was adopted for deposition. Electron beam evaporation was used to grow Au nanohelices on a BK7 glass substrate. In the deposition process, the substrate normal was tilted at an angle of 86° to the direction of incidence of the vapor. Liquid nitrogen was passed through a loop underneath the substrate to cool the substrate holder to − 140 °C. The deposition rate was maintained at 0.3 nm/s. Three substrate spin rates of 0.088, 0.117, and 0.160 rpm were chosen to match the deposition rate. Figure 2 shows cross-sectional and top-view scanning electron microscopic (SEM) images of the three 2-turn Au nanohelix array. Table 1 presents the pitch length and radius of curvature of the three samples. The nanohelix arrays (sample 1 and sample 2) that were deposited at spin rates of 0.088 and 0.117 rpm were spiral-like. As the spin rate increased from 0.088 to 0.117 rpm, the pitch length decreased from 70 to 60 nm and the radius of curvature decreased from 45 to 30 nm. The mean size of the helices that were grown on the seeded surface was successfully reduced from the previously deposited Au nanohelices, with a pitch length of 162 nm and a helix radius of 78 nm that were grown on a smooth glass surface [12, 13]. The nanohelix array (sample 3) that was deposited at the spin rate of 0.160 rpm was screw-like, and its mean pitch length of 55 nm is smaller than that of sample 2. Furthermore, the mean radius of curvature of sample 3 is reduced to be 17 nm. A 2-turn Au nanohelix array deposited at a spin rate of 0.117 rpm is also shown in Fig. 2g, h. It is demonstrated that the Au nanohelixes are failed to grow on a smooth substrate.

**Table 1:** Radius of curvature and pitch length of the three samples

	Spin rate(ω) (rpm)	Radius of curvature (*R*) (nm)	Pitch length (*P*) (nm)
Sample 1	0.088	45	70
Sample 2	0.117	30	60
Sample 3	0.16	17	55

**Fig. 3:**
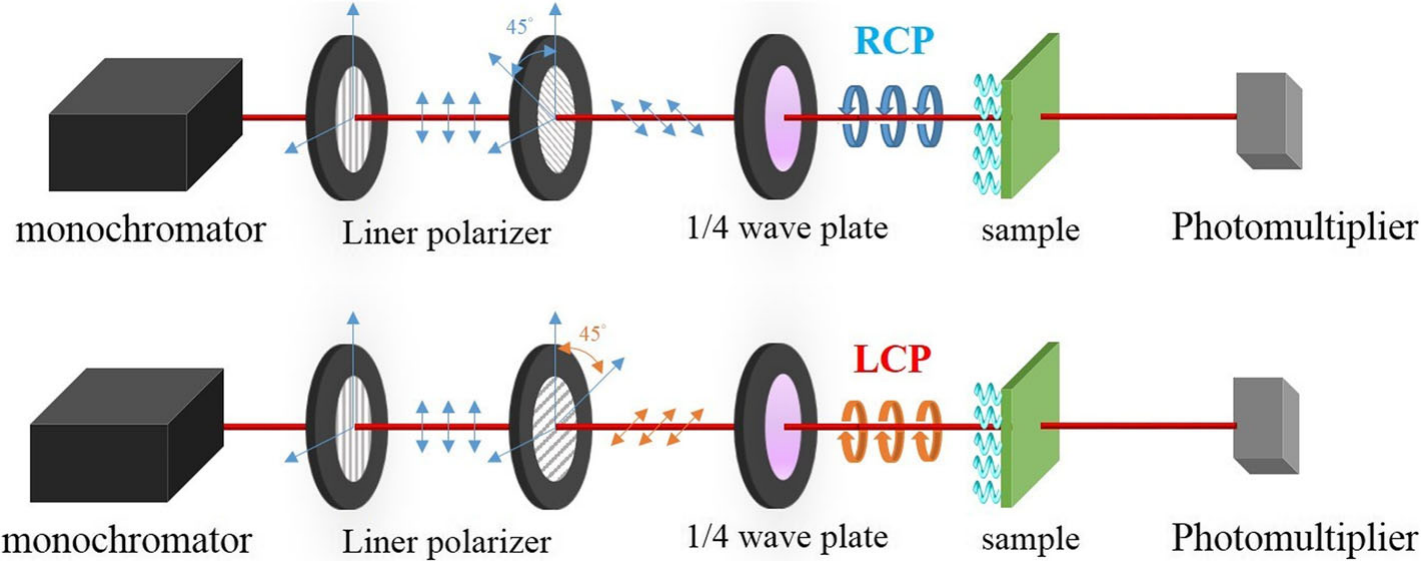
Schematics of the experimental setup for optical measurements

In our measurement, we applied a linear polarizer and an achromatic waveplate in front of the light source to generate circular polarized waves with wavelengths from 400 to 700 nm. The measurement setup is added in Fig. 3. The transmittance and reflectance spectra associated with right-handed and left-handed incident light are measured to derive extinctance spectra. The sample was rotated and stopped every 45° to measure the reflectance and transmittance spectra at the eight different orientations. It was found that the measured spectra are extremely low dependent on the orientation of rotation; the difference of transmittance or reflectance values between any two orientations is less than 0.167%. The circular dichroism of the sample was measured as the g-factor (*g*), which is defined by the equation,$g=\left({E}_{\mathrm{RCP}}-{E}_{\mathrm{LCP}}\right)/\left(\frac{\left({E}_{\mathrm{RCP}}+{E}_{\mathrm{LCP}}\right)}{2}\right)$ where the extinctance *E*_RCP_ (*E*_LCP_) was measured by illuminating the sample with right-handed (left-handed) circular polarized light. The extinctance *E* is defined as *E* = 1 − *R* − *T* where *R* and *T* are reflectance and transmittance, respectively.

## Results and Discussion

**Fig. 4:**
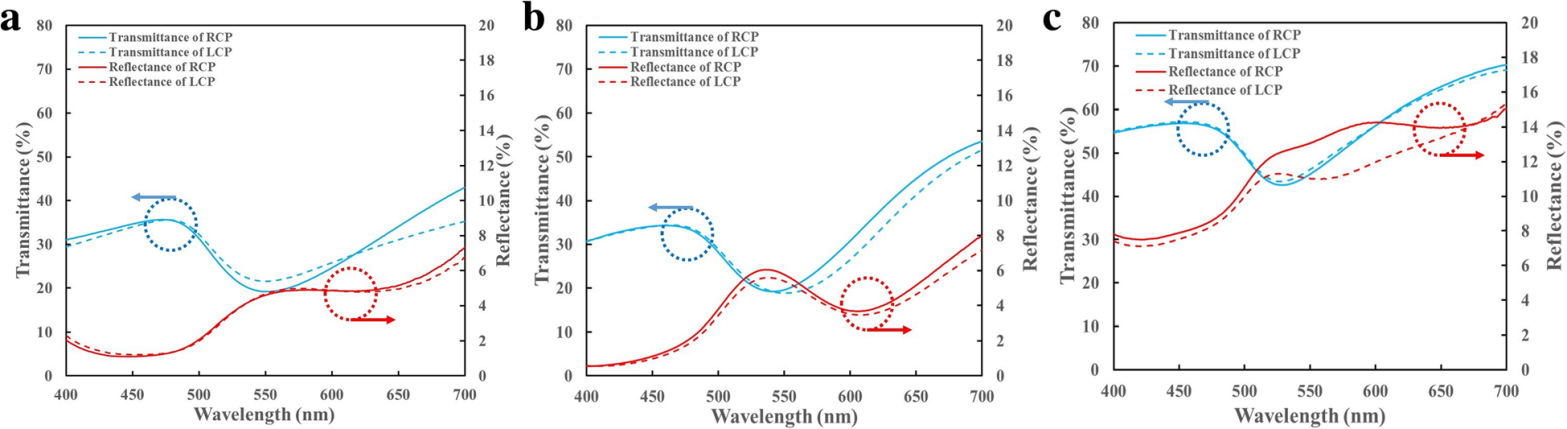
Right-handed and left-handed circular polarized transmittance and reflectance spectra of sample 1 (**a**), sample 2 (**b**), and sample 3 (**c**)

 Figure 4 shows the transmittance and reflectance spectra for both circular polarization states. The two spiral-like samples have similar spectra, with a transmittance dip and a reflectance peak at wavelengths between 500 and 600 nm. The transmittance of screw-like sample 3 exceeds that of the other two spiral-like samples, and its reflectance remains higher than 8% in the visible regime. At wavelengths between 400 and 700 nm, the transmittance values of both polarization states are higher than 43%.

**Fig. 5:**
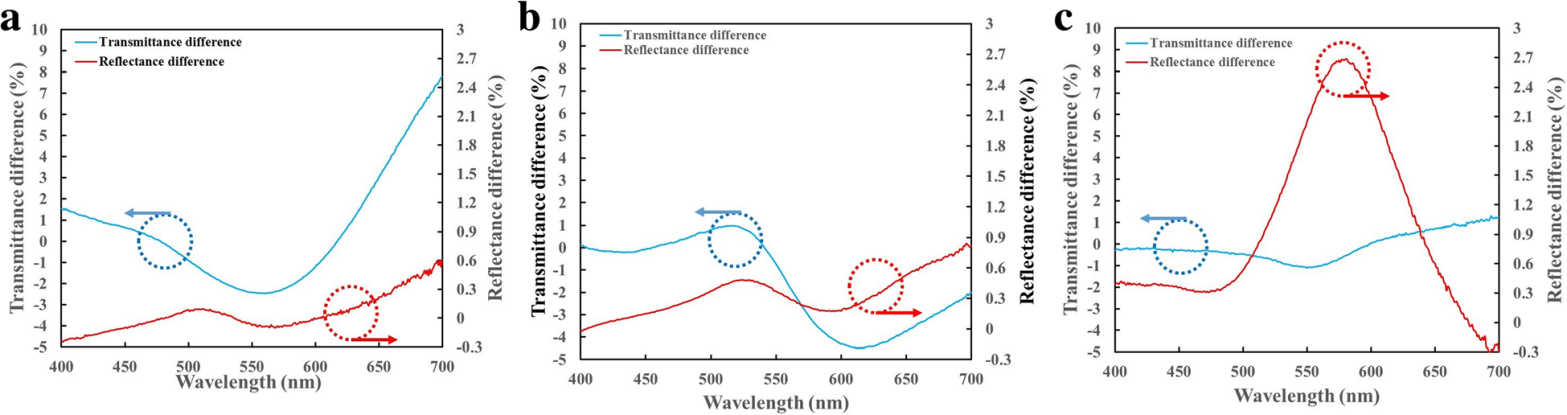
Transmittance difference (Δ*T*) and reflectance difference spectra (Δ*R*) of sample 1 (**a**), sample 2 (**b**), and sample 3 (**c**)

Figure 5 shows the spectra of transmittance difference and reflectance difference between right-handed and left-handed circular polarization states. For sample 1, the transmittance difference Δ*T* = *T*_RCP_ − *T*_LCP_ decrease from 1.54% at λ = 400 nm to 2.47% at λ = 560 nm and then increase to 7.78% at λ = 700 nm, as shown in Fig. 5a. The reflectance difference Δ*R* = *R*_RCP_ − *R*_LCP_ is less than 0.61% in the visible regime. The maximal reflectance is 7.35% for RCP at 700 nm and 6.74% for LCP at λ = 700 nm. For sample 2, the transmittance difference Δ*T* = *T*_RCP_ − *T*_LCP_ increases from 0.13% at λ = 400 nm to 0.98% at λ = 515 nm and then decreases to − 4.48% at λ = 617 nm, as shown in Fig. 5b. The reflectance difference Δ*R* = *R*_RCP_ − *R*_LCP_ is less than 0.87% in the visible regime. The maximal reflectance is 7.99% for RCP and 7.17% for LCP at λ = 700 nm. For sample 3, the transmittance of both polarization states are very similar. The transmittance difference Δ*T* = *T*_RCP_ − *T*_LCP_ less than 1.25% in the visible regime, as shown in Fig. 5c. The reflectance difference Δ*R* = *R*_RCP_ − *R*_LCP_ rises from 0.38% at λ = 400 nm to maxima 2.68% at λ = 581 nm and drops to − 0.3% at λ = 700 nm.

**Fig. 6:**
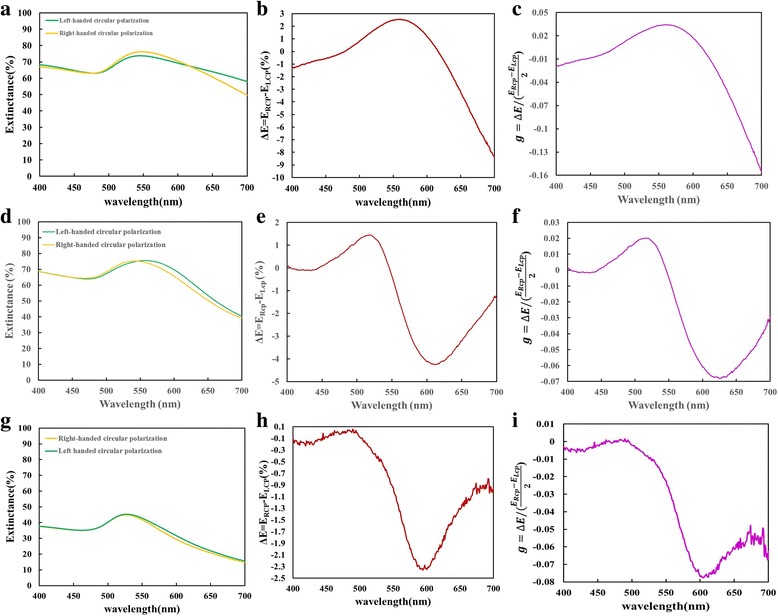
Experimental extinctance, extinctance difference (Δ*E*), and the g-factor spectra of sample 1 (**a**–**c**), sample 2 (**d**–**f**), and sample 3 (**g**–**i**)

Figure 6 shows the extinctance, extinctance difference (Δ*E* = *E*_RCP_ − *E*_LCP_), and g-factor spectra. For the sample 1, the maximum of extinctance difference Δ*E*_max_ *=* 2.56% occurs at λ = 560 nm and the minimum of extinctance difference Δ*E*_min_ *=* −8.39% at λ = 700 nm. The g-factor is within the range between 0.0344 and − 0.156 over wavelengths from 400 to 700 nm. The g-factor reaches its extreme values at λ = 560 nm (*g* = 0.034) and λ = 700 nm (*g* = −0.156). For the sample 2, the maximum of extinctance difference Δ*E*_max_ *=* 1.45% occurs at λ = 517 nm and the minimum of extinctance difference Δ*E*_min_ *=* −4.26% at λ = 612 nm. The g-factor is in the range from 0.02 to − 0.068 at wavelengths from 400 to 700 nm. The extreme values of the g-factor are reached at λ = 517 nm (*g* = 0.02) and λ = 617 nm (*g* = −0.068). For the sample 3, the extinctance difference is small and below 0.055%. A localized g-factor maximum at λ = 490 nm is 0.00146, and a localized g-factor minimum at λ = 605 nm is − 0.07768. For the three samples, the g-factor maximum shifts from 560 to 490 nm as the radius of curvature of the nanohelices is reduced from 45 to 17 nm.

**Fig. 7:**
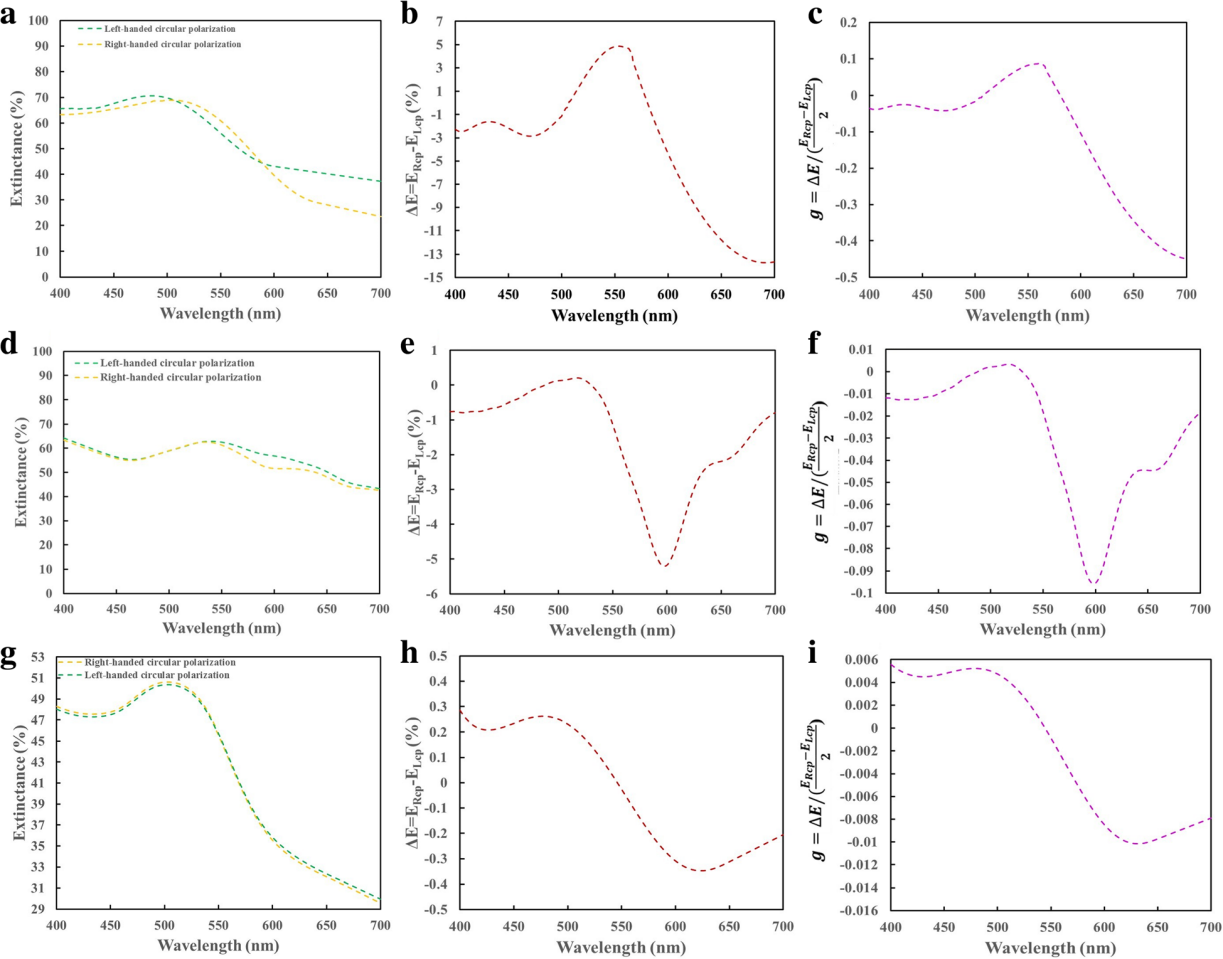
Simulated extinctance, extinctance difference (ΔE), and the g-factor spectra of sample 1 (**a**–**c**), sample 2 (**d**–**f**), and sample 3 (**g**–**i**)

**Fig. 8:**
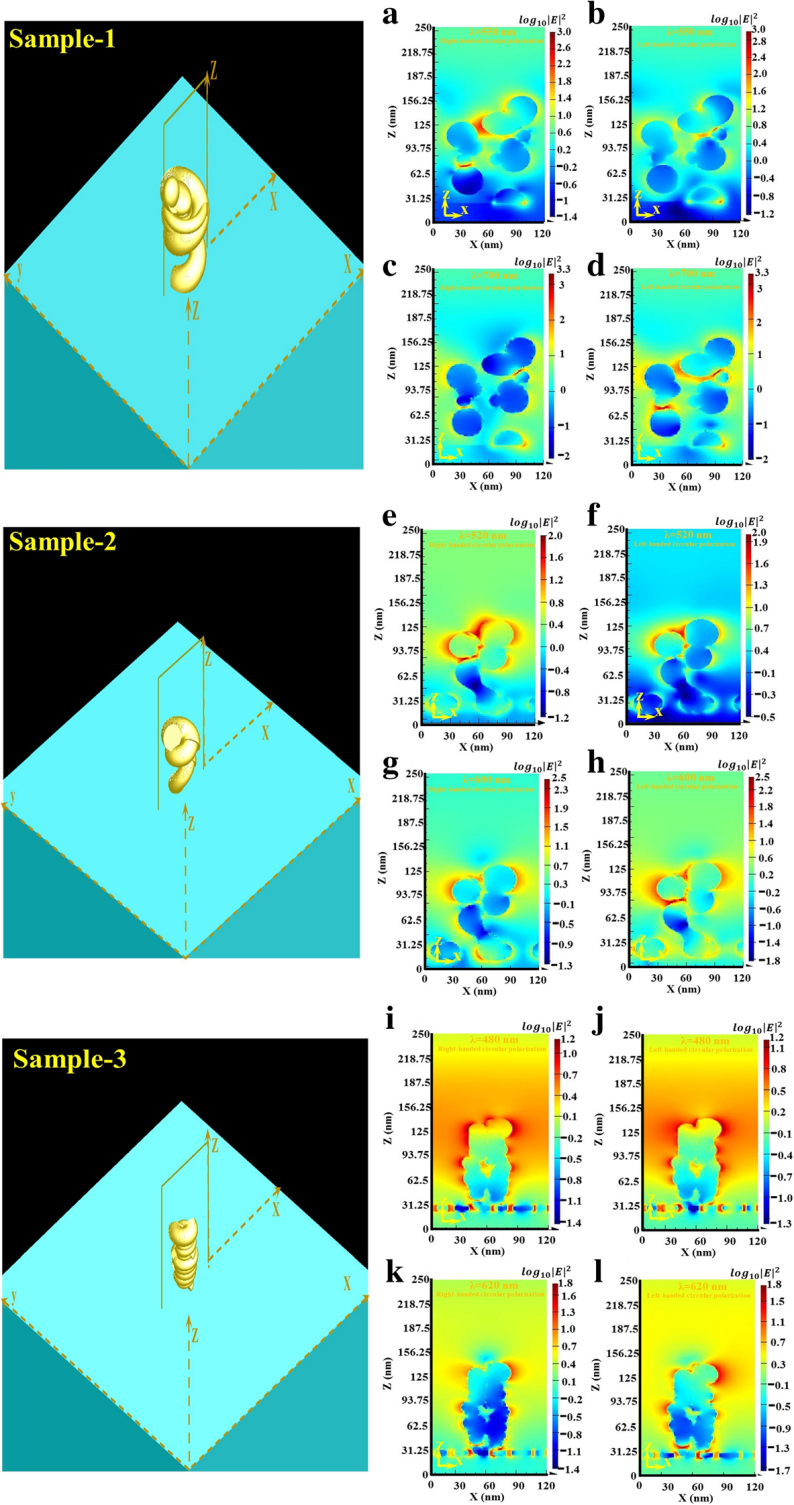
Schematic diagrams of Au nanohelices and electric field intensity distribution of sample 1 (**a**–**d**), sample 2 (**e**–**h**), and sample 3 (**i**–**l**)

The relationship between the morphology of Au nanohelix array and extinctance spectra is investigated with near field simulation. 3-D finite-difference time-domain (FDTD) simulations (Lumerical FDTD Solutions 8.7.11) are performed. The set parameters for the FDTD calculations include a 1-nm mesh and time step of 0.001 fs. The permittivity of gold was adopted from Johnson and Christy in the material library of the software [18]. The aforementioned average pitch length, radius of curvature, and spacing of fabricated gold nanohelixes are adopted to build the gold nanohelix array for simulation. The simulated extinctance, extinctance difference (Δ*E*), and g-factor spectra of the three arrays of helixes are shown in Fig. 7. The simulation results are quantitatively in agreement with the measurement results. On the other hand, the wavelength λ_max_ corresponding to the positive maximum g-factor and the wavelength λ_min_ corresponding to the negative minimum g-factor are adopted to simulate the near field distribution. The (λ_max_, λ_min_) of sample 1, sample 2 and sample 3 are (550 nm, 700 nm), (520 nm, 600 nm), and (480 nm, 620 nm), respectively. The right-handed (left-handed) circular polarized light waves with wavelength of λ_max_ and λ_min_ are normally incident onto the sample and the electric field intensity defined as |*E/E*_*i*_|^2^ where *E* and *E*_*i*_ are the amplitudes of localized electric field and incident electric filed, respectively, are simulated for its distribution on the Au nanohelix array. Figure 8 shows the field intensity distribution on the cross-section (xz-plane) for each sample. For each sample, it is obvious that the localized field intensity under RCP illumination is stronger than that illuminated with LCP light at the wavelength of λ_max_. On the other hand, the localized field intensity under LCP illumination is stronger than that illuminated with RCP light at the wavelength of λ_min_. The magnitude difference of maximum local field intensity between RCP and LCP illumination is obvious for sample 1 and sample 2. For sample 3, the localized field intensity distributions of both polarization states are very similar. The near field simulation can explain the measured results qualitatively.

## Conclusions

In conclusion, a surface on which particles are distributed has been formed by annealing an ultra-thin metal film. The particles have a shadowing effect in glancing angle deposition and influence the size of nanohelices that are grown on them. The substrate spin rate was tuned relative to the deposition rate to mass-produce spiral-like and screw-like nanohelices with a feature size of less than 100 nm. The near field simulation is adopted to explain the polarization-dependent extinctance. The demonstrated size-dependent circular dichroism enables the fabrication of nanohelices with designated chiral optical property.

## Abbreviations

FDTD: Finite-difference time-domain
LCP: Left-handed circular polarized
RCP: Right-handed circular polarized
SEM: Scanning electron microscopic
